# Ecological diversity of a snake assemblage from the Atlantic Forest at the south coast of Paraíba, northeast Brazil

**DOI:** 10.3897/zookeys.787.26946

**Published:** 2018-10-02

**Authors:** Ivan L. R. Sampaio, Claudileide P. Santos, Rafaela C. França, Isabella M. M. C. Pedrosa, Mirco Solé, Frederico G. R. França

**Affiliations:** 1 Programa de Pós-graduação em Ecologia e Monitoramento Ambiental, Universidade Federal da Paraíba – UFPB, Av. Santa Elizabete, s/n – Centro, CEP 58297-000 Rio Tinto, PB, Brazil; 2 Programa de Pós-graduação em Ciências Biológicas – Zoologia, Universidade Federal da Paraíba, Castelo Branco, CEP 58051-900 João Pessoa, PB, Brazil; 3 Programa de Pós-graduação em Ecologia e Conservação da Biodiversidade, Universidade Estadual de Santa Cruz, Rodovia Jorge Amado, Km 16, CEP 45662-900 Ilhéus, Bahia, Brazil; 4 Herpetology Section, Zoologisches Forschungsmuseum Alexander Koenig, Adenauerallee 160, 53113 Bonn, Germany; 5 Núcleo de Ecologia de Serpentes UFPB, Laboratório de Ecologia Animal, Universidade Federal da Paraíba – UFPB, CCAE, Av. Santa Elizabete, s/n – Centro, CEP 58297-000 Rio Tinto, PB, Brazil; 6 Departamento de Ciências Biológicas, Universidade Estadual de Santa Cruz, Rodovia Jorge Amado, km,16, 45662-900 Ilhéus, Bahia, Brazil; 7 Departamento de Engenharia e Meio Ambiente, Centro de Ciências Aplicadas e Educação, Universidade Federal da Paraíba – UFPB, Av. Santa Elizabete, s/n – Centro, CEP 58297-000 Rio Tinto, PB, Brazil

**Keywords:** inventory, natural history, open formations, restinga, Serpentes, species richness

## Abstract

Despite an increase in studies focusing on snake ecology and composition in the northeastern Atlantic Forest, several poorly studied sites and environments remain. The aim of this study was to assess species richness and natural history attributes of the snakes of an assemblage in the Restinga, Tabuleiro and Forest environments of the Atlantic Forest of the south coast of Paraíba, northeastern Brazil. A total of 151 individuals of 27 species, 23 genera, and six families of snakes were found. The most effective sampling methods were time-constrained searches and incidental encounters. Species sampled most frequently were the blindsnake *Epictiaborapeliotes*, the Boa Constrictor *Boaconstrictor*, the Brown Vinesnake *Oxybelisaeneus*, and the Brazilian False Coral Snake *Oxyrhopustrigeminus*. The snake fauna is characterized mainly by terrestrial species found in open-area environments of Restinga and Tabuleiro, and with most species feeding on amphibians and small mammals. The rarefaction curve did not reach the asymptote and new species should be recorded for south coast of Paraíba in future studies. Despite the richness and composition of snakes of the south coast being similar to other areas in the state, there is a lack of some species typically linked to forests, and this is probably because of the high level of deforestation that the south area of the state has suffered.

## Introduction

Biological surveys are the foundation for our knowledge on biodiversity. They also provide the groundwork for ecological studies and provide an outline for implementing conservation strategies (Greene 1994, Sutherland 1997). Since 1990, there has been an increase in surveys of Brazilian snakes, with information about richness and natural history published for different biomes, such as Amazonia (Martins and Oliveira 1998, Bernarde and Abe 2006), Southeast Atlantic Forest (Marques and Sazima 2004, Pontes et al. 2009), Northeast Atlantic Forest (França et al. 2012, Dias and Rocha 2014, Marques et al. 2016, Roberto et al. 2017), Caatinga (Vitt and Vangilder 1983, Mesquita et al. 2013), Cerrado (França et al. 2008, França and Braz 2013), Pantanal (Strussmann and Sazima 1993, Strüssmann et al. 2011), and Pampas grasslands (Winck et al. 2007). However, some sites and environments are still poorly known, such as coastal restingas and mangroves. Although some studies have been carried out in the restingas areas of the southeast of Brazil and in the State of Bahia in the northeast of Brazil (e.g. Rocha 2000, Hamdan and Lira-da-Silva 2012, Santos et al. 2012, Dias and Rocha 2014, Marques et al. 2016), no study was conducted in these environments in the most septentrional portion of the Atlantic Forest of the Northeast.

The Atlantic Forest is the most threatened biome in Brazil, and it has been suffering from intense deforestation and fragmentation since the time of European colonization. Even though less than six percent of the original vegetation remains, the Atlantic Forest still harbors high levels of biodiversity with more than 8,000 endemic species of vascular plants, amphibians, reptiles, birds and mammals (Myers et al. 2000). This degradation is even more evident in the portion of the Atlantic Forest located north of the São Francisco River, where an important endemism center in South America, the Pernambuco Endemism Center (hereafter PEC), is situated (Prance 1982, Tabarelli et al. 2005). In this region, sugarcane is the main agricultural crop, and other anthropogenic actions, such as animal and vegetal extraction, have reduced biodiversity in this region (Coimbra-Filho and Câmara 1996, Dean 1996). Today, the remaining 2% of the original forest cover of the PEC is represented by small fragments (mostly less than 10 ha) inserted in urban and agricultural matrices (Brown and Brown 1992, Ranta et al. 1998, Silva and Tabarelli 2000). The high richness and extensive threat of Northeast Atlantic Forest’s loss underscore the urgency to increase our knowledge of this region’s biodiversity to develop effective conservation plans for the biome (Tabarelli et al. 2005).

In the Atlantic Forest of the four states (Rio Grande do Norte, Paraíba, Pernambuco and Alagoas) that comprise the PEC, the four main vegetation physiognomies in the region are: (a) Mangroves, saline-adapted tropical vegetation on the coast; (b) Coastal Restingas, low forest that grows on coastal sand dunes; (c) Semi-deciduous Forests, also known as Lowland Tabuleiro Forests, that are evergreen forests with natural savanna enclaves (called tabuleiros) that occur over faster-draining sand soils, and (d) Highland Stationary Forests, that are humid forest remnants scattered throughout Caatinga Highlands, known as Brejos de Altitude (Oliveira-Filho and Carvalho 1993, Thomas and Barbosa 2008).

The snake fauna of the Atlantic Forest of Paraíba State, in Northeast Brazil, has been studied previously, in the central and north portions, but the south of the State remains unknown (Pereira-Filho et al. 2017). Herein, we describe the snake composition at the south coast of Paraíba, providing information on richness and natural history attributes of the species, and comparing it with other snake assemblages of the region.

## Materials and methods

**Figure 1.**
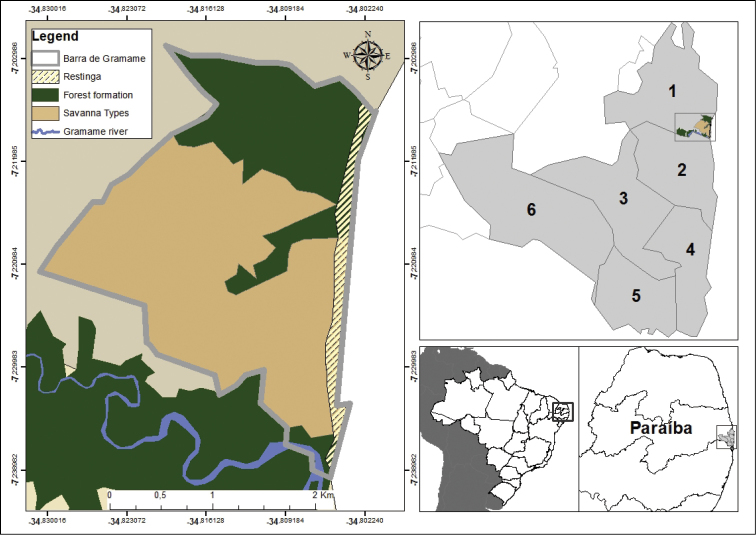
Schematic map showing the south coast of Paraíba state. Detail box: Barra de Gramame, located in the municipality of João Pessoa. The numbers represent the following municipalities: **1** João Pessoa **2** Conde **3** Alhandra **4** Pitimbu **5** Caaporã and **6** Pedras de Fogo.

The study was conducted in Barra de Gramame, located in the south of the municipality of João Pessoa at the South Coast of Paraíba State, Northeast Brazil (Figure 1) (07°14'00.5"S, 34°48'21.6"W; SAD69). In addition, we include specimens previously collected and housed in CHUFPB (Herpetological Collection of Universidade Federal da Paraíba) from adjacent municipalities of south coast of Paraíba State. These specimens were added for better characterization of snake fauna of the region. The municipalities that compose the south coast are Alhandra, Caaporã, Conde, Pedras de Fogo, Pitimbu, and the south of João Pessoa (Figure 1). The south coast extends for 33 km in Paraíba State.

**Figure 2.**
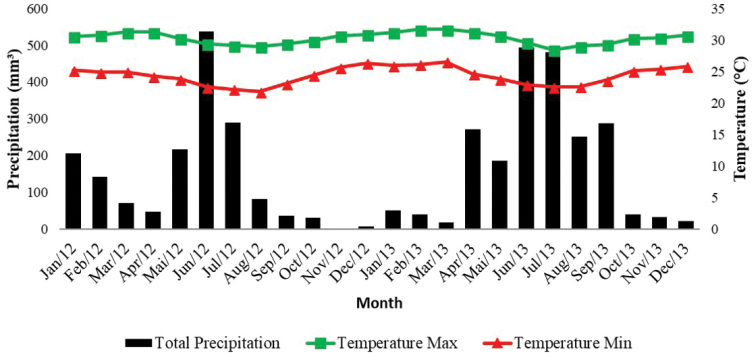
Variation of temperature, maximum (line with square) and minimum (line with triangle), precipitation as bars. Data from January 2012 and December 2013 (Source: INMET, http://www.inmet.gov.br/portal/index.php?r=bdmep/bdmep).

**Figure 3.**
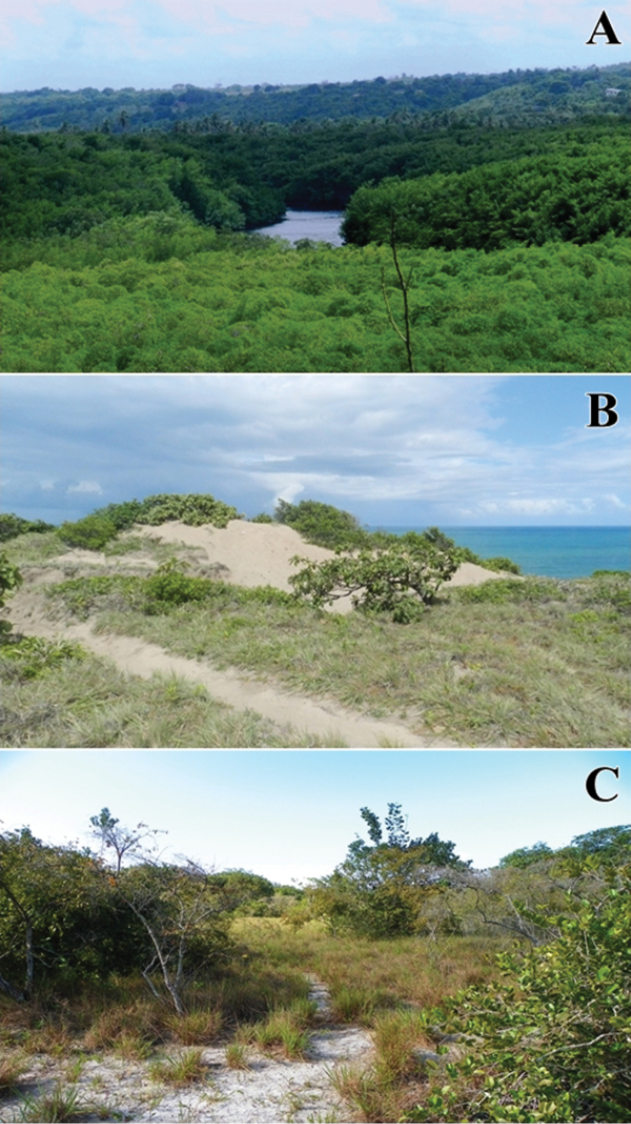
Phytophisiognomies of Atlantic Forest in south coast of Paraíba. **A** Forest **B** Restinga **C** Savanna enclave (Tabuleiro). Photograph credits: Ivan L. Sampaio (**A, B**), Frederico G. França (**C**).

The Barra de Gramame area covers approximately 843 ha (Figure 1), with mean annual precipitation of 1,800 mm and a rainy season between May and August, according to the data provided by Instituto Nacional de Meteorologia (INMET, http://www.inmet.gov.br/portal/index.php?r=bdmep/bdmep) (Figure 2). Surveys were conducted from 1 January 2012 through 31 December 2013 in all three physiognomies present in the area: Evergreen Forests and Savanna enclaves – both from Lowland Tabuleiro Forests – and in Coastal Restingas (Figure 3).

For the fieldwork conducted in Barra de Gramame, we employed the following snake survey methods: time-constrained search, incidental encounters, and specimens donated by local people. Snake specimens were obtained from January 2012 to December 2013 predominantly through 2520 man-hours of time-constrained search (see Martins and Oliveira 1998) in both diurnal (1620 men/h) and nocturnal (900 men/h) surveys. Regarding the donated specimens, we did not encourage local people to kill snakes, but they usually kill all encountered snakes, so we asked them to preserve the dead snakes for further identification. The collection methods for specimens of other municipalities (not Barra de Gramame in João Pessoa) were not available in the Herpetological Collection.

For each specimen found in Barra de Gramame, we recorded information on location (with GPS), time, habitat use and microhabitat, and morphology. Snakes were collected under ICMBio collecting permit (SISBIO license 37318-1), preserved in 10% formalin and deposited in Coleção Herpetológica da Universidade Federal da Paraíba (Appendix). Some individuals of the most common species were released after we marked them by clipping ventral scales to identify them as previously captured. No recapture was obtained in this study. We categorized the snake size considering the mean body size of each species based on published data (França and Araújo 2006, Pereira-Filho et al. 2017) as small (<500mm), moderate (501–1000mm) and large (>1001mm).

We constructed the species accumulation curves for the snakes of the South Coast using the individual-based rarefaction method (with the nonparametric Mao Tau estimator) to evaluate the relationship between collection effort and species saturation in the assemblage (Gotelli and Colwell 2001). The function of richness (Mao Tau) was calculated as the accumulation function of species throughout the number of individuals collected. The species rarefaction curve was made without replacement using 1000 randomizations. In addition, we used species richness estimators (with nonparametric incidence-based estimators: Bootstrap, Chao 2, ICE, Jacknife 1 and 2, and abundance-based data: ACE and Chao 1) to determine the expected richness of snakes in each area (Colwell and Coddington 1994). The species rarefaction and richness estimators were performed with EstimateS 9.1.0 software (Colwell 2013).

## Results

**Figure 4.**
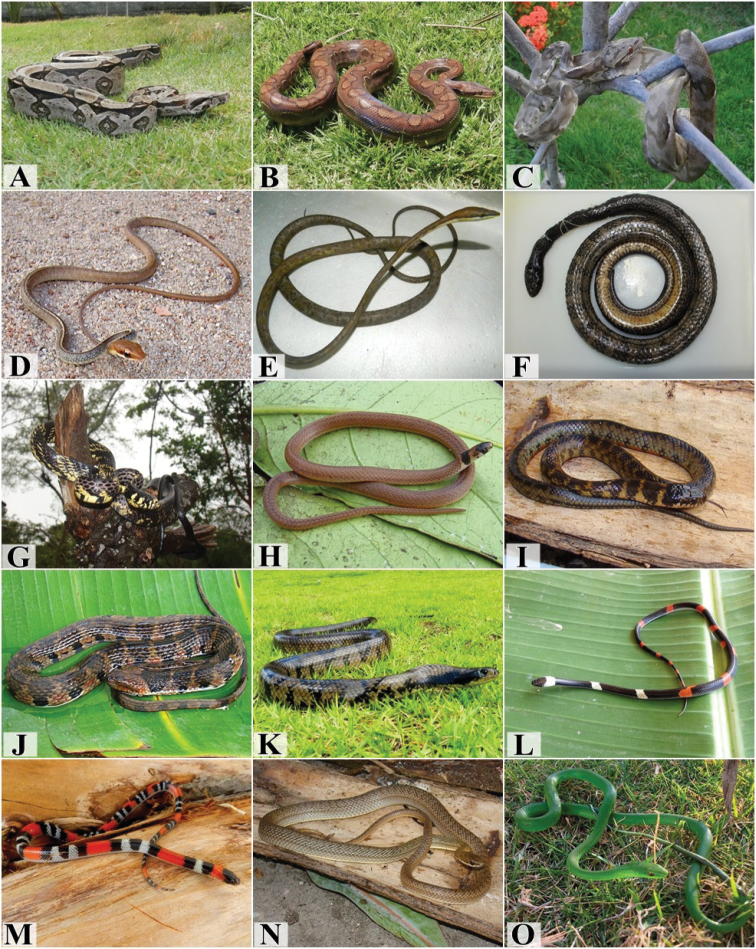
Species from the Atlantic Forest in south coast of Paraíba. **A***Boaconstrictor***B***Epicratesassisi***C***Corallushortulanus***D***Chironiusflavolineatus***E***Oxybelisaeneus***F***Mastigodryasbifossatus***G***Spilotespullatus***H***Tantillamelanocephala***I***Erythrolamprustaeniogaster***J***Helicopsangulatus***K***Hydrodynastesgigas***L***Oxyrhopuspetolarius***M***Oxyrhopustrigeminus***N***Philodryasnattereri***O***Philodryasolfersii*. Photograph credits: Ivan L. Sampaio (**A, B, C, E, K, L, M, N, O**), Frederico G. França (**D, H, I, J**), Mayara Morais (**F**), Willianilson Pessoa (**G**).

**Figure 5.**
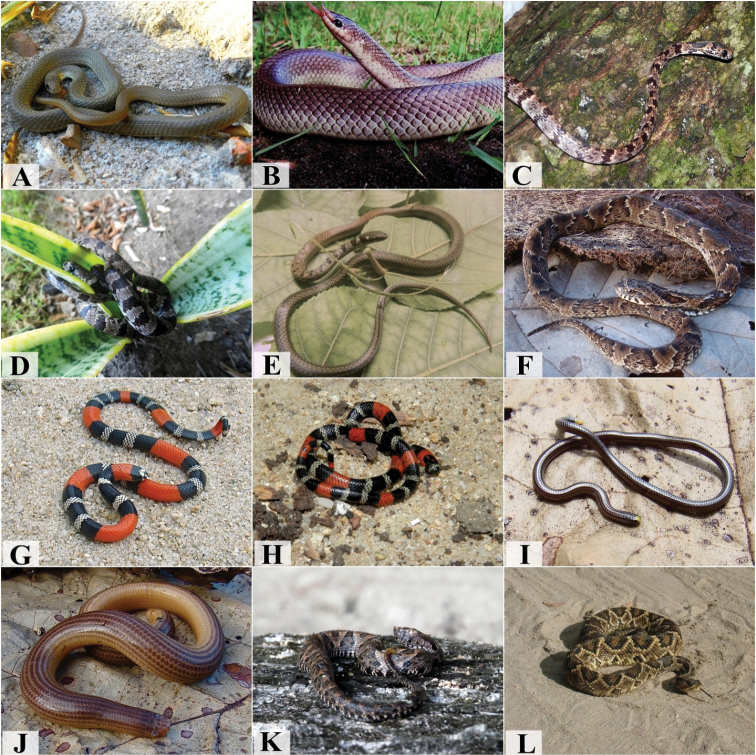
Species from the Atlantic Forest in south coast of Paraíba: **A***Philodryaspatagoniensis***B***Phimophisguerini***C***Sibonnebulatus***D***Sibynomorphusmikanii***E***Taeniophallusoccipitalis***F***Xenodonmerremii***G***Micrurusibiboboca***H***Micruruspotyguara***I***Epictiaborapeliotes***J***Amerotyphlopsbrongersmianus***K***Bothropsleucurus***L***Crotalusdurissus*. Photograph credits: Ivan L. Sampaio (**A, B, D, E, H, I**), Frederico G. França (**F, G, J, L**), Pedro T. S. Moura (**C**) Rafaela C. França (**K**).

We recorded 151 individuals of 27 species, 23 genera, and six families (Boidae, Colubridae, Elapidae, Leptotyphlopidae, Typhlopidae, and Viperidae) (Table 1, Figures 4, 5). The most common snake species were the blindsnake *Epictiaborapeliotes*, the Boa Constrictor *Boaconstrictor*, the Brown Vinesnake *Oxybelisaeneus*, the Brazilian False Coral Snake *Oxyrhopustrigeminus*, and the Patagonia Green Racer *Philodryaspatagoniensis* representing more than 50% of all records (Table 1). The rarest species were the Garden Tree Boa*Corallushortulanus*, Boettger’s Sipo *Chironiusflavolineatus*, the Rio Tropical Racer *Mastigodryasbifossatus*, the Caninana *Spilotespullatus*, the watersnake *Erythrolamprustaeniogaster*, the Forest Flame Snake *Oxyrhopuspetolarius*, the Argentine Pampas Snake *Phimophisguerini*, Wagler’s Snake *Xenodonmerremii*, the Caatinga Coral Snake *Micrurusibiboboca*, and Brongersma’s Worm Snake *Amerotyphlopsbrongersmianus*, with only one record each. All sample methods contributed to the snake sampling. Of the 118 individuals found in Barra de Gramame, 52 were captured during time-constrained searches, local collectors donated 13 individuals, and 48 were found by incidental encounters.

**Table 1.** Summary of the Information of Natural History of the Snakes in Barra de Gramame. Abbreviations of Municipalities are: AL = Alhandra; CA = Caaporã; CO = Conde; JP = João Pessoa (Barra de Gramame), PF = Pedras de Fogo, PI = Pitimbu. Habits are: AB = arboreal, AQ = aquatic, CR = cryptozoic, FO = fossorial, TE = terrestrial, SAB = semi-arboreal. Habitats are: T = tabuleiro, F = forest, R = restinga, * = Data from Herpetological Collection. Diet are: am = amphibians, an = anfisbaenas, ar = arthropods, bi = birds, fi = fishes, li = lizards, ma = mammals, sl = snails, sn = snakes. Capture Methods are: TCS = time-constrained search, DO = donated, IE = incidental encounters; – = No data available (see methods).

Family / Species (number of species)	N	Municipality	Habits	Habitats	Diet	Capture method
**Boidae (3)**						
*Boaconstrictor* Linnaeus, 1758	16	JP	SAB	T, F, R	ma, bi	TCS, DO, IE
*Corallushortulanus* (Linnaeus, 1758)	1	JP	AB	T	ma, bi	DO
*Epicratesassisi* Machado, 1945	10	AL, CO, JP, PI	TE, SAB	T, F	ma, bi, li	TCS, DO
**Colubridae; Colubrinae (5)**						
*Chironiusflavolineatus* Jan, 1863	1	AL	SAB	F*	am	–
*Oxybelisaeneus* (Wagler, 1824)	14	JP	AB	T	li	TCS, DO, IE
*Mastigodryasbifossatus* (Raddi, 1820)	1	CO	TE	T*	ma	–
*Spilotespullatus* (Linnaeus, 1758)	1	PF	SAB	F*	ma, bi	–
*Tantillamelanocephala* (Linnaeus, 1758)	5	AL, JP	FO	T, R	ar	TCS, IE
**Colubridae; Dipsadinae (13)**						
*Erythrolamprustaeniogaster* (Jan, 1863)	1	JP	TE, AQ	R	fi, am	IE
*Helicopsangulatus* (Linnaeus, 1758)	3	CA, JP	AQ	R	fi, am	TCS
*Hydrodynastesgigas* (Duméril, Bibron & Duméril, 1854)	5	JP	AQ	R	fi, am, sn, ma	TCS, IE
*Oxyrhopuspetolarius* Reuss, 1834	1	JP	TE	T	ma, li	TCS
*Oxyrhopustrigeminus* Duméril, Bibron & Duméril, 1854	16	CA, CO, JP	TE	T, F	ma, li	TCS, DO, IE
*Philodryasnattereri* Steindachner, 1870	8	AL, JP	TE	T	ma, bi, li, an	TCS, DO, IE
*Philodryasolfersii* (Lichtenstein, 1823)	10	JP, PI	TE, SAB	T	ma, bi, li, an	TCS, DO, IE
*Philodryaspatagoniensis* (Girard, 1858)	11	JP	TE	T, R	ma, bi, li, an	TCS, DO, IE
*Phimophisguerini* (Duméril, Bibron & Duméril, 1854)	3	CO, JP	CR, TE	T	li	IE
*Sibonnebulatus* (Linnaeus, 1758)	1	PF	AB	F	sl	–
*Sibynomorphusmikanii* (Schlegel, 1837)	10	AL, CA, JP	TE	T	sl	DO
*Taeniophallusoccipitalis* (Jan, 1863)	7	AL, CA, JP	TE	T	am	TCS, DO, IE
*Xenodonmerremii* (Wagler, 1824)	1	CO	TE	F*	am	–
**Elapidae (2)**						
*Micrurusibiboboca* (Merrem, 1820)	1	AL	CR, TE	T*	an, sn	–
*Micruruspotyguara* Pires, Silva, Feitosa, Prudente, Pereira Filho & Zaher, 2014	3	JP	CR, TE	T	an, sn	TCS, DO
**Leptotyphlopidae (1)**						
*Epictiaborapeliotes* (Vanzolini, 1996)	16	JP	FO	T	ar	TCS, DO, IE
**Typhlopidae (1)**						
*Amerotyphlopsbrongersmianus* (Vanzolini, 1976)	1	CA	FO	T*	ar	–
**Viperidae (2)**						
*Bothropsleucurus* Wagler, 1824	2	AL, CA	TE	F*	ma, li, an	–
*Crotalusdurissus* Linnaeus, 1758	2	CO	TE	R*	ma	–

**Figure 6.**
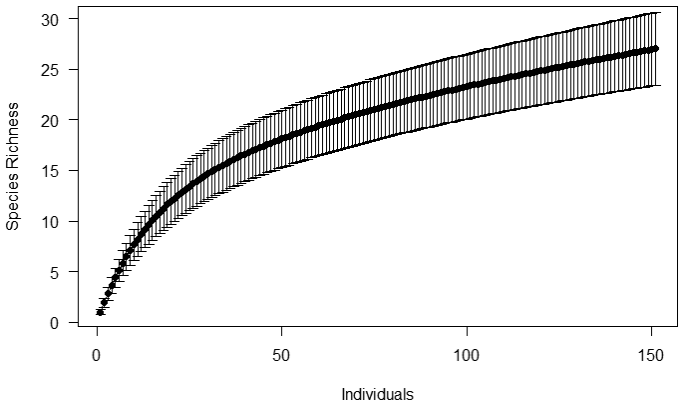
Individual-based rarefaction curve with standard deviation of snake species of south coast of Paraíba.

The individual-based rarefaction curves didn’t reach stability (Figure 6) and the species richness estimators produced estimates greater than the observed richness, indicating a higher number of species for the locality. Estimates varied between 31.09 ± 0.35 and 51.83 ± 32.04 (Table 2).

**Table 2.** Richness estimators of snake assemblages for the south coast of Paraíba.

Richness Estimators	Mean ± Std Deviation
Observed Richness	27
ACE	40.59 ± 1.16
CHAO 1	51.83 ± 24.08
CHAO 2	51.83 ± 32.04
ICE	40.21 ± 1.13
JACKNIFE 1	36.93 ± 3.05
JACKNIFE 2	44.84 ± 0.91
BOOTSTRAP	31.09 ± 0.35

The snakes showed a diversity of habits and food preferences (Table 1). Three species are fossorial (11%), three are primarily cryptozoic (11%), three show aquatic habits (11%), ten are strictly terrestrial (37%), five utilize both terrestrial and (semi-) arboreal habits (18%), and three are strictly arboreal snakes (11%). Of the 27 snake species, 22 were found exclusively in one habitat of the south coast: 13 in Tabuleiro, four in Restinga, and five in Forest. In addition, two were found in Tabuleiro and Restinga, two in Tabuleiro and Forest, and only *Boaconstrictor* was found in all habitats (Table 1).

### Natural history of species

Below we present information on morphology, habitat use, and activity of all snake species found on the south coast of Paraíba. We include data on diet, habitat, and habits from published literature.

BOIDAE: *Boaconstrictor* – A large terrestrial species (average SVL = 904.25 mm; range from 400 to 2000 mm; N = 16). Second most common species in the study area. Six individuals were found active during the day between 10:40 and 17:30, despite literature records indicating nocturnal activity for the species (Henderson and Powell 2007). Four snakes showed aggressive behavior during the approach, striking and opening their mouth. Other three individuals were found dead by local people. This species eats mammals and birds (Marques et al. 2001); *Corallushortulanus* – A large arboreal snake (SVL = 945 mm; N = 1). The species was found resting in the roof of a house at 15:30 and was striking constantly during the capture. This species eats mammals and birds (Marques et al. 2001); *Epicratesassisi* – A large terrestrial species (average SVL = 788 mm; range from 560 to 1240 mm; N = 5). All individuals were collected alive and active at night (from 18:00 to 19:25) both in Tabuleiro and Forest. This species eats mammals, birds and lizards (Vitt and Vangilder 1983).

COLUBRIDAE: COLUBRINAE: *Chironiusflavolineatus* – A moderate-sized semi-arboreal species (SVL = 598 mm; N = 1). No data of habitat use for the south coast is available. This species eats primarily amphibians (Pereira-Filho et al. 2017). *Oxybelisaeneus* – An arboreal, moderate-sized species (average SVL = 721 mm; range 550 to 985 mm; N = 14). All individuals were found active during the day (from 7:40 to 16:00) and most (11) were in trees and shrubs, while three were found active on the ground. Some individuals presented open-mouth threats and striked during the approach, while others remained immobile, swaying their body like a branch. In addition, many individuals displayed cloacal discharge during handling. This species eats primarily lizards (França and Araújo 2007); *Mastigodryasbifossatus* – A large terrestrial species (SVL = 325 mm; N = 1). No data of habitat use for the south coast is available. This species eats amphibians and small mammals (Marques and Muriel 2007). *Spilotespullatus* – A large, semi-arboreal species (SVL = 580 mm; N = 1). No data of habitat use for the south coast is available. This species eats primarily mammals and birds (Pereira-Filho et al. 2017). *Tantillamelanocephala* – A small-sized terrestrial species (average SVL = 193 mm; range 150 to 210 mm; N = 4). One individual captured in tabuleiro and one found dead on unpaved road. Two individuals were active during the afternoon (16:00–16:30), although the species is known to forage at night for active prey, mainly centipedes (Marques and Puorto 1998).

COLUBRIDAE: DIPSADINAE: *Erythrolamprustaeniogaster* – A moderate-sized semi-aquatic species (SVL = 385 mm; N = 1). One individual was found resting in the water at 9:00. This species eats anurans (França et al. 2012). *Helicopsangulatus* – A moderate-sized aquatic species (SVL = 635 mm; N = 1). One individual was found in mangroves in the river. This species eats fishes and anurans (Ford and Ford 2002); *Hydrodynastesgigas* – A large aquatic species (average SVL = 884 mm; range 714 to 1048 mm; N = 5). Despite this species being considered rare in Northeast Brazil (Pereira-Filho et al. 2017), we recorded an intermediate abundance (N = 5). Local fisherman killed two individuals in the morning (approximately 8:00 and 8:30) when they were moving between sand beaches and salty water. Another two were found dead on a paved road near a river. One active individual was captured in restinga at 10:00 and displayed several defensive behaviors such as striking and biting, tail whip, and cloacal discharge. This species eats fishes, anurans, snakes and mammals (López and Giraudo 2004); *Oxyrhopuspetolarius* – A moderate-sized terrestrial species (SVL = 245 mm; N = 1). The individual found was active in tabuleiro habitat at 18:20. This species eats lizards, small mammals and birds (Cunha and Nascimento 1978). *Oxyrhopustrigeminus* – A moderate-sized terrestrial species (average SVL = 420 mm; range 174 to 900 mm; N = 12). Seven individuals were active at night (18:00–20:30), and two in the afternoon (15:50 and 17:00). Five were found moving on the ground of tabuleiro and two were immobile on leaf litter. Some individuals displayed cloacal discharge during handling. This species eats lizards and small mammals (França et al. 2008); *Philodryasnattereri* – A large-sized terrestrial (or semi-arboreal) species (average SVL = 813 mm; range from 500 to 981 mm; N = 6). All individuals were found active during the day (from 10:40 to 13:30) moving on sandy soil or near residences. Three individuals displayed aggressive behaviors of striking and biting. This species has a generalized diet, which includes mammals, birds, lizards and amphibians (Vitt and Vangilder 1983); *Philodryasolfersii* – A moderate-sized semi-arboreal species (average SVL = 557 mm; range from 410 to 750 mm; N = 9). Seven individuals were found active during the day (from 10:00 to 15:20). Four snakes displayed aggressive behaviors of striking, biting and cloacal discharge. This species eats anurans, small mammals, lizards and birds (Hartmann and Marques 2005, Leite et al. 2009); *Philodryaspatagoniensis* – A moderate-sized terrestrial species (average SVL = 576 mm; range from 250 to 885 mm; N = 11). Six individuals were found active during the day (from 10:00 to 13:00), and all displayed defensive behaviors of striking, biting and cloacal discharge. This species has a generalized diet, which includes mammals, birds, lizards and amphibians (Hartmann and Marques 2005); *Phimophisguerini* – A moderate-sized terrestrial species (SVL = 687 mm; N = 1). Only one individual was found active in the twilight (17:50) moving on a paved road. It eats lizards (Sawaya et al. 2008). *Sibonnebulatus* – A small to moderate size arboreal species (SVL = 400mm; N=1). One individual was found active at 13:00 in a forest edge. This species eats snails (Pereira-Filho et al. 2017); *Sibynomorphusmikanii* – A small to moderate size terrestrial snake (average SVL = 287 mm; range from 155 to 420 mm; N = 2). One individual was found inactive on the floor of a house and one was active at night near a small pond. This species eats snails (Laporta-Ferreira et al. 1986). *Taeniophallusoccipitalis* – A small to moderate size terrestrial species (average SVL = 295 mm; range from 150 to 450 mm; N = 5). Four individuals were found active during the day (from 15:00 to 17:30) in tabuleiro habitat. This species eats amphibians (Marques et al. 2001). *Xenodonmerremii* – A large size terrestrial species (SVL = 388 mm; N = 1). No data of habitat use for the south coast is available. This species eats frogs (Pereira-Filho et al. 2017).

ELAPIDAE: *Micrurusibiboboca* – A moderate-sized cryptozoic species (SVL = 525 mm; N = 1). No data of habitat use for the south coast is available. This species eats amphisbaenians and snakes (Roze 1996). *Micruruspotyguara* – A moderate-sized cryptozoic species (average SVL = 634 mm; range 320 to 925 mm; N = 3). Three individuals found active at night (from 19:00 to 21:00) and near residences. Two snakes displayed defensive behaviors of tail raising and head hiding. This species eats amphisbaenians and snakes (Pereira-Filho et al. 2017).

LEPTOTYPHLOPIDAE: *Epictiaborapeliotes* – A small-sized fossorial species (average SVL = 110 mm; range from 70 to 136 mm; N = 16). This was the most common species in Gramame and it was very adapted to anthropic areas. All individuals were found active moving on sand, on grass and on unpaved and paved roads, during the day and twilight (from 8:45 to 17:30). Many individuals stung with tail points when handled. The representatives of this family eat ants and larvae, but there is no information on the diet of this species.

TYPHLOPIDAE: *Amerotyphlopsbrongersmianus* – A small-sized fossorial species (SVL = 212 mm; N = 1). No data of habitat use for the south coast is available. This species eats ant larvae (Ávila et al. 2006).

VIPERIDAE: *Bothropsleucurus* – A large terrestrial species (SVL = 587 mm; N = 1). This species frequently is found in the urban area and in preserved forest patches. No data of habitat use for the south coast is available. This species eats frogs (juveniles) and small mammals (adults) (Campbell and Lamar 2004). *Crotalusdurissus* – A large terrestrial species (SVL = 1070 mm; N = 2). No data of habitat use for the south coast is available. This species eats small mammals (Campbell and Lamar 2004).

## Discussion

The snake fauna of Barra de Gramame presents similar richness and composition compared with other snake assemblages from central and north coast of Paraíba State (Pereira-Filho et al. 2017). The snake fauna of the south coast comprises species found in other areas of Atlantic Forest of Paraíba State in the north coast (Pereira-Filho et al. 2017). However, some common species were absent and should appear in future surveys, such as the Pernambuco Worm Snake *Typhlopspaucisquamus* and the Yellow-bellied Swamp Snake *Erythrolampruspoecilogyrus*. Nevertheless, some snakes that are rare at the north coast, such as the False Water Cobra *Hydrodynastesgigas*, and the Garden Tree Boa*Corallushortulanus*, were found in the area and represent new distribution records for the State (Pereira-Filho et al. 2017).

Despite the savanna enclaves extending along all the Northeast Atlantic Forest, this physiognomy is poorly known and highly threatened because of agriculture and pastures (Endres et al. 2007, Thomas and Barbosa 2008). Nevertheless, these open environments can harbor high diversity and a rich snake fauna, with species found in other open biomes, such as Cerrado and Caatinga, but not in forest portions of Atlantic Forest (Rodrigues et al. 2015. Fossorial species (such as scolecophidians) are commonly found in these environments, mainly because of the sandy soil (Rodrigues et al. 2015).

Although the reptiles of costal Restingas have been commonly studied in the Southeast and Central Atlantic Forest (Rocha 2000, Santos et al. 2012, Marques et al. 2016), little information is available for northern portions of the biome, and here we present the first records of snakes from coastal Restingas of Paraíba State. We found some similarities of composition in this habitat with both *Boaconstrictor* and *Tantillamelanocephala* that also commonly are found in southeastern Restingas (Dias and Rocha 2014, Martins et al. 2012), and aquatic snakes found both in fresh and salty water. The Cascabel Rattlesnake *Crotalusdurissus* is also found in other Restingas (Marques et al., 2016). This species is associated with open areas, commonly found in Cerrado and Caatinga (França et al. 2008; Vanzolini et al. 1980) and it supposedly reaches Brazilian coast regions dispersing through deforested areas of Atlantic Forest (Bastos et al. 2005).

Two species are typical of forest environments (*Sibonnebulatus* and *Bothropsleucurus*), and six are habitat generalist, occurring also in open areas. This richness of forest snake species for the south coast is low if compared with assemblages studied for the north and central coasts (Pereira-Filho et al. 2017). While we found only 20% of snakes inhabiting forests of the south coast, the snake fauna of north and central coast of the state are represented by more than 50% of forests snake species (Pereira-Filho et al. 2017).

Despite the presence of open area of Restinga and Tabuleiros, there is a lack of large forest patches in the south coast that are present in other areas of Paraíba State (Rodrigues et al. 2015). Most of the forest in the south coast has been lost or reduced to small fragments since the occupation of the area for agriculture and urbanization (Furrier and Silva Barbosa 2016). Therefore, the snake fauna seems to be depleted, lacking some species typically linked to forests, such as *Imantodescenchoa*, *Thamnodynasteshypoconia*, *Pseustessulphureus*, and *Lachesismuta*. The open areas of restinga and tabuleiro still are preserved more than forests, reflecting in more species found in these locations. However, the region of Gramame, in the south of João Pessoa municipality, and other south coast beaches, such as Coqueirinho and Tambaba, in Conde municipality, and Praia Bela, in Pitimbu municipality are parts of a tourist occupation program of the south coast of Paraíba, which should increase the damage both on vegetation and on the Gramame river basin (Silva et al. 2002).

It is important to emphasize that there are large gaps in the conservation status of snake species in the PEC region; of the 27 species of snakes reported in this study, only two species (*Corallushortulanus* and *Crotalusdurissus*) were evaluated by the IUCN. In addition, some species are still being discovered in the PEC region, and have little information available, such as *Micruruspotyguara* (described in 2014) (Pires et al. 2014) and *Amerothyplopsarenensis* (described in 2015) (Graboski et al. 2015).

Finally, comparing with other snake assemblages in Paraíba, the fauna of south coast shows some peculiarities. The most common species was a blindsnake, the same as the North Coast (Rodrigues et al. 2015). However, the species differ in the areas (*Epictiaborapeliotes* in Gramame vs *Typhlopspaucisquamus* in Guaribas Biological Reserve). The scolecophidian snakes are small and fossorial species, and the sandy soil of restinga and tabuleiro forests may favor the presence of these species. Also, we recorded the presence of two rare species for Paraíba state, the False Water Cobra *Hydrodynastesgigas* and the Garden Tree Boa*Corallushortulanus*. These species have only been found in few areas in Paraíba (Pereira-Filho et al. 2017), and the former seems to be threatened in the State, mostly because of the high levels of water pollution in rivers where the species is present (Pereira-Filho et al. 2017). Additional species are expected to appear in future surveys in Gramame and in the south coast of Paraíba. The information acquired from snake species composition, ecology and distribution should be used for the planning of urbanization and conservation of the south coast of Paraiba.

## Appendix 1

Snake species collected at the Paraíba state’s south coast, northeast Brazil, and housed at the Coleção Herpetológica da Universidade Federal da Paraíba

**João Pessoa – Barra de Gramame**: *Boaconstrictor* (CHUPB 12252/RT 419, RT 603, RT 661, RT 790), *Corallushortulanus* (BG 40), *Epicratesassisi* (RT 792), *Epictiaborapeliotes* (RT 531, RT 645, RT 646, RT 705, RT 825, RT 864, RT 887, RT 890), *Erythrolamprustaeniogaster* (RF 106), *Helicopsangulatus* (RT 847), *Hydrodynastesgigas* (BG 26), *Micruruspotyguara* (RT 860), *Oxybelisaeneus* (RT 606, RT 700, RT 747, RT 861, RT 862, RT 889), *Oxyrhopuspetola* (RT 701), *Oxyrhopustrigeminus* (RT 703, RT 765, RT 793, RT 859, RT 891, RT 892), *Philodryasnattereri* (CHUFPB 7669/RT 166, RT 763), *Philodryasolfersii* (RT 072, RT 702, RT 748, RT 888), *Philodryaspatagoniensis* (RT 223, RT 604, RT 605, RT 706, RT 750, RT 791), *Taeniophallusoccipitalis* (RT 704, RT 749), *Tantillamelanocephala* (RT 766, RT 893); **Alhandra**: *Bothropsleucurus* (UFPB 12963); *Chironiusflavolineatus* (UFPB 9374); *Epicratesassisi* (UFPB 13163), *Micrurusibiboboca* (UFPB 13636); *Philodryasnattereri* (UFPB 13642); *Sibynomorphusneuwiedi* (UFPB 12643); *Taeniophallusoccipitalis* (UFPB 9390); *Tantillamelanocephala* (UFPB 9389); **Caaporã**: *Amerotyphlopsbrongersmianus* (CHUFPB 15704); *Bothropsleucurus* (UFPB 12961); *Helicopsangulatus* (CHUFPB 15670, 15715); *Oxyrhopustrigeminus* (CHUFPB 15806); *Sibynomorphusmikanii* (UFPB 12948-53); *Taeniophallusoccipitalis* (CHUFPB 15837); **Conde**: *Crotalusdurissus* (UFPB 5053, 5803); *Epicratesassisi* (UFPB 13163), *Mastigodryasbifossatus* (UFPB 4825); *Oxyrhopustrigeminus* (UFPB 13157-8); *Phimophisguerini* (UFPB 13976-7); *Xenodonmerremii* (UFPB 13974); **Pitimbu**: *Epicratesassisi* (UFPB 13164, 13179, 13199), *Philodryasolfersii* (UFPB 13650); **Pedras de Fogo**: *Spilotespullatus* (UFPB 13981).
